# Noncovalently assembled nanotubular porous layers for delaying of heating surface failure

**DOI:** 10.1038/srep06817

**Published:** 2014-10-29

**Authors:** Bong June Zhang, Taeseon Hwang, Jae-Do Nam, Jonghwan Suhr, Kwang Jin Kim

**Affiliations:** 1 Active Materials and Smart Living (AMSL) Laboratory, Mechanical Engineering Department, University of Nevada, Las Vegas, Nevada 89154, USA; 2 NBD Nanotechnologies, 8 saint Mary's street, Boston, Massachusetts 02215, USA; 3 Department of Polymer Science & Engineering, Sungkyunkwan University, 300 Chunchun-dong, Jangan-gu, Suwon, Gyeonggi-do, 440-746, South Korea

## Abstract

Thermal management to prevent extreme heat surge in integrated electronic systems and nuclear reactors is a critical issue. To delay the thermal surge on the heater effectively, we report the benefit of a three dimensional nanotubular porous layer via noncovalent interactions (hydrophobic forces and hydrogen bonds). To observe the contribution of individual noncovalent interactions in a porous network formation, pristine carbon nanotubes (PCNTs) and oxidatively functionalized carbon nanotubes (FCNTs) were compared. Hydrogen-bonded interwoven nanotubular porous layer showed approximately two times critical heat flux (CHF) increase compared to that of a plain surface. It is assumed that the hydrophilic group-tethered nanotubular porous wicks and enhanced fluidity are the main causes for promoting the CHF increase. Reinforced hydrophilicity assists liquid spreading and capillarity-induced liquid pumping, which are estimated by using Electrochemical Impedance Spectroscopy. Also, shear induced thermal conduction, thermal boundary reduction, and rheology of nanoparticles could attribute to CHF enhancement phenomena.

Preventing extreme thermal energy surge is an important parameter to design electronic devices and nuclear reactors in power plants to avoid emergent shut down and/or damage. Beyond a certain thermal limit, sudden wall superheat triggers individual bubble coalescence and vapor column formation on the heating surface and leads to heater failure. This phenomenon is called the CHF, which is directly related to the safe operation of thermal energy conversion systems^1^. The majority of research in CHF has been focused on heating surface wetting^2^,^3^,^4^,^5^. Enhanced surface wetting can delay CHF and/or augment the CHF limit by supplying liquid and spreading it into a hot spot effectively. Recently, several research groups demonstrated significant CHF enhancement and wall superheat reduction using artificially engineered nano porous surfaces^6^,^7^,^8^,^9^,^10^,^11^,^12^,^13^.

Also, various nanofluid approaches have been introduced throughout the last decade. The high thermal conductivity and enhanced surface wetting through nanofluid-deposited porous heating surfaces are known to contribute to significant CHF improvement^13^,^14^,^15^,^16^,^17^,^47^. Among the various nanofluids, those with carbon nanotubes (CNTs) have been considered as ideal candidates due to CNT's outstanding thermophysical properties and mechanical robustness^18^,^19^,^20^,^21^.

In this report, we present increase in the CHF using a noncovalently self-assembled carbon nanotubular porous layer and demonstrate its plausible mechanism through various experimental investigations. Also, we highlight how the CHF enhancement phenomena is attributed to shear induced thermal conduction and thermal boundary reduction.

## Results

**Figure 1:**
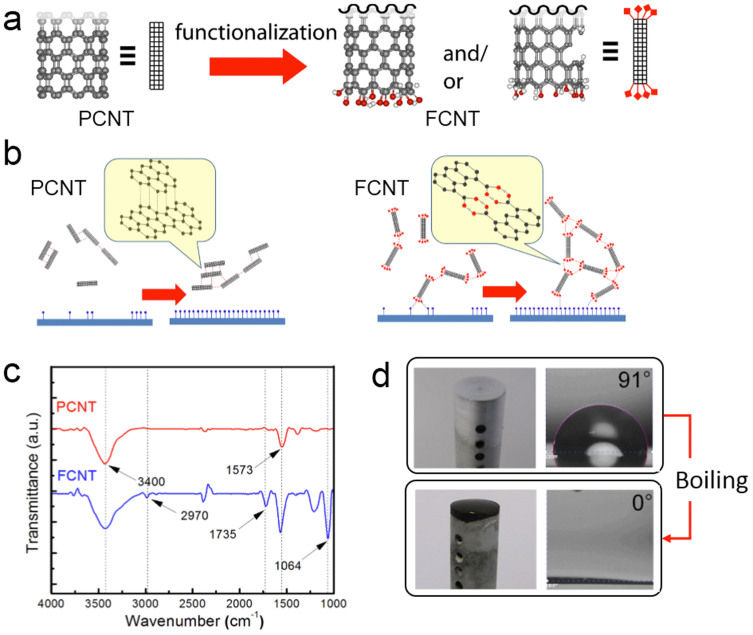
Schematic illustration of functionalized-CNT (FCNT) deposition and photographs of aluminum heating surfaces: (a) Acid catalyst-assisted functionalization of PCNT yields FCNT: ball and stick model. Black, red, and white ball represent carbon, hydrogen, and oxygen, respectively. (b) Schematic illustration of individual PCNTs (left) and FCNTs (right). For PCNTs, major driving force of aggregation is hydrophobic forces. For FCNTs, numerous hydrogen bonds induce agglomerate formation and easily anchor on the aluminum substrate (Al_2_O_3_ → Al_2_(OH)_3_). (c) FT-IR spectra of PCNTs (red) and FCNTs (blue), and (d) Before boiling, mirror-finished aluminum heater surface is shown (left). Inset shows apparent contact angle of water (91°). After boiling, black FCNT porous surface, which enhances instantaneous liquid wetting, is seen on right. Inset shows apparent contact angle of water (~0°). Heater diameter is 1 cm.

As schematically illustrated in Figure 1a, PCNTs (black ball represents carbon atom) were oxidized by acid treatment to functionalize with carboxylic group (red and white ball represent oxygen and hydrogen atoms, respectively)^22^,^23^,^24^,^25^,^26^,^27^,^28^. During the oxidation process, CNTs are partially damaged (broken walls) and nano pores are formed. Further oxidation causes CNT ends to be carboxylic-tethered and/or fused to each other as shown arrows in Figure S1a. The carboxylic group is a key moiety, which leads to hydrogen bonding interactions in an aqueous fluid (polar water). Scanning electron microscope (SEM) images of PCNTs show the individually segmented shape in Figure S1b. Nonpolar PCNTs aggregate in aqueous fluid via hydrophobic forces compared to hydrogen bonds of FCNTs^29^.

To depict nanotubular porous layer formation process, schematic illustration is shown in Figure 1b. Nonpolar PCNTs are not miscible in water (a polar protic solvent). Water molecules near the PCNT aggregates are prone to rearrange to compensate disrupted hydrogen bonds. Entropically unfavorable new configuration of water leads to the merging of adjacent hydration shells, which are long ranged van der Waals interaction^30^. In order to reduce the energy penalty, PCNTs aggregate. The overlapping of hydration shells is the driving force for the self-assembly of PCNTs. This phenomenon is often observed in the self-assembly of biological membranes^31^, protein folding^32^, polymer association^33^, and micellization^34^.

On the contrary, FCNTs do not have a solvation issue due to the hydrogen-tethered carboxylic groups, which can lead to entropically favorable self-assembly in an aqueous fluid. Carboxylic groups preorganize via inter- and/or intramolecular hydrogen bonds depending on molecular distance and configuration. Simultaneously, they form bifurcative hydrogen bonds with water molecules, which assist salvation. As temperature increases, the aluminum substrate begins to oxidize (Al_2_O_3_ → AlOOH, Al(OH)_3_ are, red dots, depicted on the aluminum substrate)^35^. The hydroxy group of aluminum hydroxide plays the role as a hydrogen bonding site. Carboxylic groups of FCNTs form hydrogen bonding networks with the alcohol group of the heating surface. Once FCNT aggregates are nucleated on the aluminum surface at a higher temperature, they build up layers and form nanotubular porous surface. Cooperativity in hydrogen bondings between aluminum and FCNTs enhances bond strength and leads FCNTs to anchor onto the heating surface with ease as shown in Figure 1b^36^.

After functionalization of PCNTs, FCNTs have a huge structural change, which can be characterized by using FT-IR spectroscopy. As shown in Figure 1c, the peak at 1,573 cm^−1^ is attributed to the carbon double bonding (C = C) in the hexagonal structure of CNTs and FCNTs^37^. The broad band in both specimens at a range of 3,100–3,600 cm^−1^ was attributed to -OH stretching vibrations from hydroxyl groups and residual adsorbed water during preparation of KBr pellet^38^. There are several convincing peaks for carboxylic functional groups of the FCNTs in the FT-IR spectrum of FCNTs. The sharp peak at 1,064 and 1,735 cm^−1^ corresponding to the C-O asymmetric and symmetric stretching vibration and the C = O stretching vibration of carboxyl groups, respectively^38^,^39^. Appearance of peak at 2,970 cm^−1^ assigns C-H asymmetric and symmetric stretching vibration^40^.

To highlight the nanotubular porous layer formation, aluminum heater images are compared in Figure 1d. Before pool boiling, the mirror-polished aluminum heater is a shiny metallic (left in Figure 1d). Without capillary wicks (prior to FCNT treatment), no significant water spreading was observed and apparent water contact angle (AWCA) of water was close to 90°. As pool boiling reaction proceeds, built in FCNT layers lead to color change (black deposits). Due to hydrophilic porous layers, the surface was instantaneously wetted and the measured AWCA was nearly 0°.

**Figure 2:**
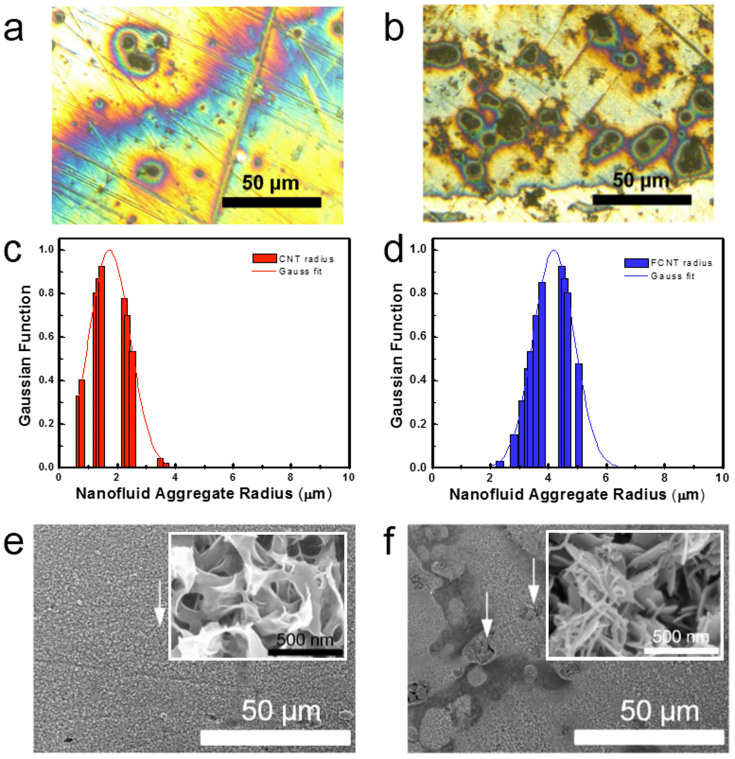
Digital microscope and FE-SEM images (×1,000 magnification) of nanotubular aggregate deposited on aluminum heater substrates: An aliquot of nanofluids was taken under reflux conditions: (a) CNT 100 wppm. (b) FCNT 100 wppm. Nanotubular aggregate radius statistics: (c) CNT 100 wppm, Mean radius 1.7 ± 0.9 μm. (d) FCNT 100 wppm, Mean radius 4.2 ± 1.7 μm. (e) Aluminum surface in CNT 100 wppm and magnified view of arrowed spot (inset). (f) Aluminum surface in FCNT 100 wppm and magnified view of arrowed spots (inset). FCNTs are attached on top of the oxidized aluminum porous wicks.

In order to better understand the FCNT aggregate phenomena during boiling, the FCNT and PCNT liquids were refluxed separately for an hour at ambient pressure. An aliquot of individual solutions was taken and deposited onto the aluminum substrates (heating surface). Deposited aluminum surface was examined by a digital microscope (Keyence, VHX-2000). As shown in Figure 2a, for PCNT, aggregates are sparsely dispersed. Assuming a spherical shape, the radii of the aggregates vary from 0.5 to 3 μm. Statistics of size distribution is shown in Figure 2c. The mean radius is approximately 1.7 μm and standard deviation of the mean radius of aggregates is large (±0.9 μm). A wide standard deviation indicates the heterogeneity of nanofluids. On the contrary, for FCNT, the aggregate has the propensity to form large aggregates between individual particulates, shown in Figure 2b. The average radius of the FCNT aggregates is about 4.2 μm (±1.7 μm) as shown in Figure 2d. It is 2.5 times larger than that of the PCNT aggregates. Since FCNTs have hydrogen bonding donors from carboxylic functional groups, not only on the nanotube ends but also on the nanotube wall (see schematic illustration in Figure 1a), it is assumed that intensified hydrogen bonds between FCNT particulates lead to enhanced nanotube aggregates in the solution. It should be pointed out that, whereas hydrophobic forces are dominant in aggregation for nonpolar PCNT, hydrogen bonding interaction is prevalent in agglomerating for polar FCNT. Improved effective size of the aggregates leads to a higher chance for binding reactive sites (–OH) of the heating surface and adsorbing on the heater. Gao et al.^41^ reported Molecular Dynamics (MD) simulation results, which showed that FCNT nanofluids have stronger reactivity compared to PCNT nanofluids. Therefore, FCNT has lower reaction energy barrier due to carboxylic groups (90 kcal/mol) than that of PCNT in an aqueous solution (180 kcal/mol).

Temperature increase influences not only liquid properties but also heating surface characteristics. As boiling proceeds, the aluminum heating surface becomes oxidized (Al → Al_2_O_3_ → AlO_2_H/Al(OH)_3_)^35^. Its chemical composition change accompanies morphological change from a plain surface into amorphous pores^35^, as shown in the inset of Figure 2e. In Figure 2f, white arrows indicate that FCNT aggregates nucleate on several spots on the oxidized heating surface and grow porous structures. Due to carboxylic functional groups, FCNTs are easily attached on top of the aluminum hydroxide porous wicks through hydrogen bonds. FCNT porous wicks grow with ease through extended hydrogen bonding networks (shown in the inset of Figure 2f).

**Figure 3:**
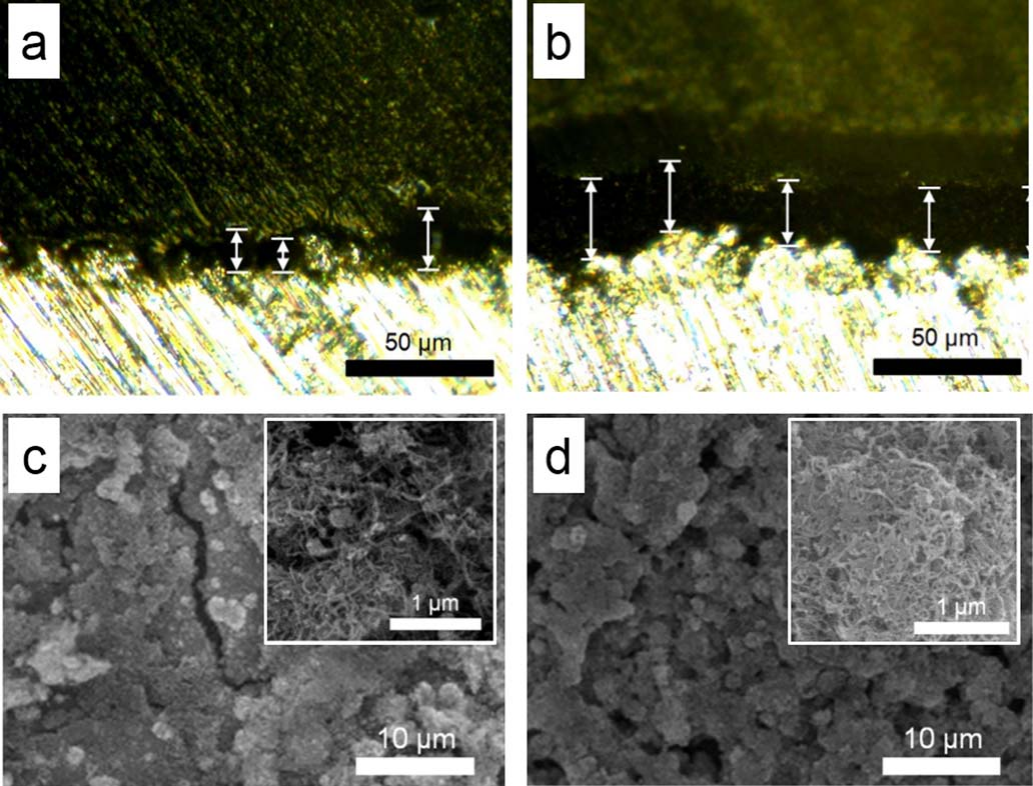
Digital microscope and FE-SEM images of nanofluids-deposited heating surfaces after pool boiling: Cross-sectional digital microscope views of deposited layers (a) PCNT on the aluminum heater.Mean thickness is 16 ± 6 μm. (b) FCNT on the aluminum heater. Mean thickness is 24 ± 2 μm. Top views of deposited layers. (c) PCNT at low magnification (×500) and at high magnification (×10k: inset). (d) FCNT at low magnification (×500) and at high magnification (×10k: inset).

In order to examine the influence of functionalized nanotube over porous matrix formation, digital microscopy and field emission-SEM (FE-SEM) images were taken from PCNT and/or FCNT-deposited heating surfaces in Figures 3 (a through d). Initially, the thickness of nanotube-deposited layer was measured from the cross-section of each heating surface. As shown in Figure 3a, for PCNT, the deposition layer is non-uniform. Some spots are rarely covered with CNT (less than 1 μm) but the other spots are densely coated (more than 15 μm) with a mean thickness of 16 ± 6 μm. Large standard deviation is associated with relatively poor hydrogen bonding interactions between CNT aggregates and the oxidized heating surface. On the contrary, for FCNT, it is notable that the deposited-layer is quite evenly distributed and a thicker layer is observed in Figure 3b with a mean thickness of 24 ± 2 μm.

Top views of each heating surfaces were taken by using FE-SEM in Figures 3 (c and d). For the PCNT-deposited layer, the size of the deposited-aggregates is irregular and some spots reveal the bare substrate (left-bottom corner in Figure 3c). A higher magnification (x 10k) FE-SEM image (inset of Figure 3c) shows that each CNT is loosely packed on the heating surface and individual CNTs are clearly seen. One thing to note is that on the CNT-deposition layer, no specific pore formation is observed. While the deposition layer of FCNT aggregates in Figure 3d seems evenly distributed. While the deposition process occurs, individual FCNT aggregates pile up and form numerous pores, which play a role of passages for water transport and facilitating water supply to the heating surface. Therefore, well-defined FCNT aggregates formation is a key to achieve efficient thermal transport during the boiling process. A higher magnification (x 10k) FE-SEM image (inset of Figure 3d) shows tightly entangled FCNT aggregates. Numerous hydrogen bondings enhance noncovalent interactions and lead to more densely packed aggregates.

The energy dispersive spectroscopy (EDS) results confirm the chemical composition of each nanofluid. In Figure S2a, there is an intensive aluminum peak (kα 1.48 keV) observed compared to the carbon peak (kα 0.28 keV) on the selected surface area (Figure S2b). This implies that there are not sufficient amounts of PCNT aggregates to cover the aluminum heating substrate thoroughly, i.e., amount of aluminum prevails over carbon atom (Al:C = 88:12). On the contrary, FCNT aggregates (see Figure 1a) lead to a high chance of binding a hydroxyl group to the aluminum heating surface. Once the FCNT aggregate nucleates on the heating surface (see arrows in Figure 2f, they propagate enormously and form densely packed porous surfaces in Figures 3(b and d). Pronounced carbon and oxygen peaks (kα 0.53 keV) in the EDS spectrum in Figure 3f validate the assumption that enhanced noncovalent interaction between the FCNTs and aluminum heating surface induces densely FCNT-coated porous wicks, i.e., atomic ratio between aluminum, carbon, and oxygen is Al:C:O = 2:75:23. Further, numerous hydrophilic FCNT-tethered pores are presumed to reinforce liquid spreading and absorption during boiling phenomena. This could be an important contribution to CHF augmentation. Gao et al.^41^ previously demonstrated enhanced wetting phenomena by MD simulations; radial density profiles of water showed a higher population density close to the carboxyl group of FCNT rather than to carbon atoms of PCNT.

**Figure 4:**
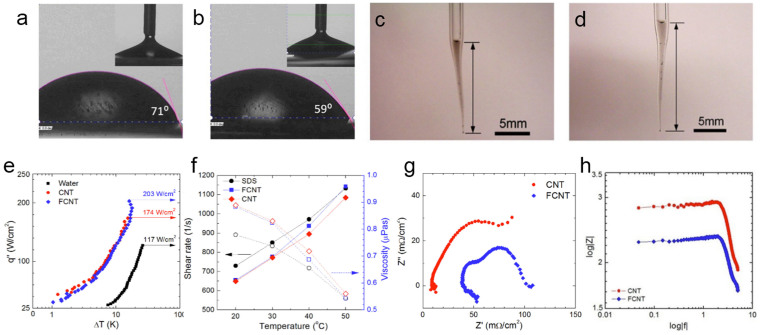
AWCA measurements at room temperature: (a) Pristine CNT on the aluminum surface shows AWCA 71° (b) FCNT on the aluminum surface shows AWCA 59°. Insets show receding angle. Capillary force measurements at room temperature: (c) Pristine CNT in the capillary (d) FCNT in the same capillary (e) Pool boiling curve (*q″* vs *ΔT*) of different fluids (water, CNT, FCNT) (f) Fluid properties of CNT and FCNT at different temperatures. Shear rate (left y-axis) and relative viscosity (right y-axis). Electrochemical impedance spectra: (g) Nyquist and (b) Bode plots of CNT-deposited aluminum slabs in 0.3 M NaCl electrolyte.

Interfacial interactions such as capillary-assisted liquid supply and liquid spreading to the dry patch on the heating surface become crucial when heat flux increases. Since wetting phenomena are closely related to noncovalent interaction (hydrogen bonding) between solid surface and liquid, FCNTs would show enhanced wetting behavior, which is a key factor to influence the CHF augmentation. To observe contribution of noncovalent interactions of the FCNTs to surface wetting, AWCA of P-/FCNT liquid was measured using a sessile drop technique on the mirror-finished reference surface (aluminum 6061, the heating surface). AWCA of PCNT liquid is approximately 71° as shown in Figure 4a. AWCA of FCNT liquid is smaller than that of PCNT liquid and shows approximately 59° in Figure 4b. Compared to the AWCA (81°) of water, almost 35% contact angle increase was observed. It implies that the combination of porous capillary wicks and FCNTs can increase substantial wetting during boiling phenomena. The improved wetting behavior of FCNT liquid at three phase interface is attributed to a carboxylic group, which has affinity to water (improved hydrophilicity). Receding AWCAs of nanofluids are shown in Figures 4 (a and b) inset. There is no substantial receding angle difference observed between PCNT and FCNT.

In addition to liquid wetting, capillary-assisted liquid supply plays an important role for thermal transport to improve CHF of nanoparticle-deposited porous media^7^,^22^. Pressure developed by capillarity is visualized and compared to each other in Figures 4 (c and d). The capillary length (*L_c,FCNT,_* ~ 17.4 mm) of the FCNT liquid in Figure 4d is longer than the CNT liquid capillary length (*L_c,CNT_* ~ 13.3 mm) in Figure 4c. It is assumed that chemically functionalized CNTs could effectively intensify capillarity-assisted liquid supply to a hot spot at high heat flux regime via enhanced noncovalent interactions.

To compare water mobility through hydrophilic functional groups in P-/FCNT-deposited layers, the electrochemical impedance spectroscopy (EIS) measurement was introduced^42^. Permeability of porous wicks as transport passages is directly influenced by the liquid diffusion. It is one of the parameters to determine the liquid-uptake quantitatively. As the electrolyte infiltration into pores proceeds, pore resistance (*R_p_*) of the porous surface gradually decreases. Simultaneously, coating capacitance (*C*) at a given time slowly increases. Individual capacitance of the porous surface is given by the Brasher-Kingsbury model^42^: 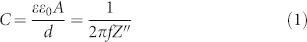
where *A*, *C*, *d*, *ε*, and *ε_0_* are exposed area, capacitance, thickness and dielectric constant of the coating, and the dielectric constant of free space, respectively. *f* and *Z″* represent frequency and impedance of the coating.

The Nyquist plot of the EIS spectrum shows a characteristic semicircle, which is a combination of each impedance value (real and imaginary impedance, *Z′* and *Z″*, respectively) in Figure 4g. The radius of the semicircle is proportional to the magnitude of impedance since impedance plays the role of the coating resistance against the electrolyte infiltration. In the Bode plot shown in Figure 4h, the impedance value of porous media is represented over a specific frequency sweep. By comparing the values obtained from the Nyquist and Bode plots at a lower frequecy regime, the contents of the electrolyte inside a porous media can be estimated. Since capacitance obtained from Eq. (1) is closely related to liquid diffusion, immersion time for all specimens is fixed at five minutes for controlled comparison purpose. Finally, water content in the porous media is calculated using Eq. (2): 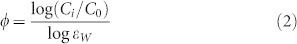
where *C_i_*, *C_0_*, and *ε_w_* are the capacitance at an instant time, *i* and initial, *0* and the dielectric constant of water, respectively. Typically, *ϕ* represents volume fraction in the coating.

FCNT aggregates have strong affinity to water through hydrogen bonds^36^. One thing to note is that the liquid absorption trend is consistent with CHF augmentation (see Figure 4e). It is assumed that the combination of hydrophilicity-reinforced surface and capillary pressure in the porous wicks would assist liquid and vapor circulation during the boiling phenomena. This can lead to substantial CHF augmentation and wall superheat reduction.

As aforementioned, noncovalently FCNTs can plays an important role in retarding heating surface failure due to improved fluidic properties compared to PCNTs. To observe the enhanced delay for the heating surface failure, critical heat flux tests of pool boiling were conducted. The pool boiling result of heat flux (*q″*) with wall superheat (*ΔT*) is presented in Figure 4e. CHF is observed at 117 W/cm^2^ for pure water. Pristine CNT liquid shows a large wall superheat reduction (~48%). CHF increases up to 174 W/cm^2^ and its enhancement ratio is ~36%. The main cause of the CHF improvement is ascribed to the thermally conductive CNT and partially CNT-deposited porous media. For FCNT, wall superheat reduction is similar to that with CNT suspended liquid. Since there is no substantial porous layer formation at the lower heat flux regime regardless if it is CNT or FCNT, heat transfer enhancement is mostly attributed due to nano particulate properties. As heat flux increases, nanoparticles aggregate with ease. They form porous layers and simultaneously, can disrupt the thermal boundary layer. This would be the main cause that delays the heating surface failure. CHF is seen at 203 W/cm^2^ (~74% enhancement). Compared to the incremental ratio of PCNT, FCNT almost doubles the effective incremental ratio.

## Discussion

Variation of CNT fluid apparent viscosity and shear rate at different temperatures is provided in Figure 4f. Viscosity of CNT fluids decreases with temperature^42^ (red diamond in Figure 4f). In general, temperature dependence of the nanofluid visocisty follows a power law correlation (*η* = *Aexp*(−*BT*) where *A* is given by 4.5–5.5 and *B* is in the range between 0.465–0.471)^41^. CNT fluid viscosity measurements are correlated with the power law. For FCNT fluids, similar trends in viscosity-temperature depedence are also observed (blue squre in Figure 4f). It is important to note is that the reduction in viscosity of FCNT fluid is somewhat obvious compared to the PCNT fluid values as temperature increases. This means shear thinning could be a contributor in FCNT fluid as temperature increases. This behavior might be attributed to the lubricating effect of nanoparticles^30^. The aggregating nanoparticles may be supporting the decrease in the friction effect and increase in lubrication^42^,^43^,^44^. As schematically illustrated in Figure 1, FCNTs are likely to agglomerate at high temperature due to their functional substituents. A larger aggregate is formed as a result (see Figure 2b). For FCNT aggregates, the effect of nanoparticle migration and rearrangement become dominant over thermal conduction^45^. Increase in effective size leads to enhanced nucleation of porous wicks. This assumption is supported by the FE-SEM image in Figure 2f^46^.

In this study, CHF enhancement phenomena utilizing CNT liquid was demonstrated. FCNTs liquid showed approximately two times CHF increase compared to that of PCNT liquid. This CHF enhancement could be attributed to improved fluid thermal conductivity and CNT-deposited porous wicks and is plausibly explained. The individual FCNT particulates self-assemble and propagate into higher order aggregates during boiling process. FCNT aggregates have several advantages in CHF enhancement: (i) improved binding affinity to the heating surfaces (ii) densely packed assembly porous wicks (iii) hydrophilicity-induced surface wetting. Well-built porous networks intensify capillarity-induced liquid pumping and spreading during the pool boiling process. In addition to the aforementioned issues, enhanced fluidity is another contributing factor, which is elucidated by shear induced thermal conduction, thermal boundary reduction, and rheology of nanoparticles.

## Methods

### Preparation of FCNT fluid

PCNTs were treated with HCl to remove impurities. CNTs (2 g) were placed in a 500 ml round bottom flask and 200 ml of HCl was added. The mixture was stirred using an overhead stirrer (Eurostar 30, IKA®) for 2 hours, then diluted in water, filtered, washed with deionized (DI) water and then dried in the convection oven at 90°C for 24 hours. In order to functionalize, 0.5 g of the purified CNTs were chemically treated in the acid solution mixture (60 ml of H_2_SO_4_, 20 ml of HNO_3_, and 1 g of KMnO_4_) in a 500 ml round bottom flask equipped with a condenser and the liquid was refluxed under an overhead stirring at 90°C for 8 hours. After that, the resulting liquid was diluted in water and filtered. The resulting solid was washed up to neutral pH, and the sample was dried in the convection oven at 90°C for 24 hours. Dried FCNTs were grinded with a mortar and pestle. The PCNTs or FCNTs were added to DI water by weight ratio (100 wppm) and dispersed with ultrasonication for an hour.

### Characterization

The FT-IR spectra of PCNTs/FCNTs were recorded between 1,000 and 4,000 cm^−1^ on a Shimadzu IRTracer-100. The PCNTs/FCNTs and dried KBr were finely grounded with a mortar and a pestle. Then, the mixture was pressed by a bolt press to form a transparent pellets. In order to measure AWCA, an ANPS heater specimen was oven-dried at 95°C for 24 hours. It was then air-cooled at ambient conditions for one hour. Using a standard syringe, a DI water sessile droplet was placed on the test specimen. All droplets were of equal volume (~5 μL). The AWCA was measured using a CAM-100 (KSV Instruments Ltd., Finland). In order to study the nanoparticle-deposited porous surfaces, the heating surface in different nanofluids (PCNTs and FCNTs) were examined by using FE-SEM and EDS chemical analysis. For CNT nanofluids, the heating surface was boiled at ambient conditions for an hour. This condition is similar to the pre-heating process of CHF experiment. The porous materials were chemically analyzed with EDS. The EDS spectrum revealed pronounced aluminum and oxide binding energy peaks at 1.48 and 0.28 keV, respectively. This implies that aluminum surfaces transformed into aluminum oxide (Al_2_O_3_).

### EIS experiment setup

A typical three electrode (test sample, reference, counter electrode) system was used to determine the EIS measurement. A carbon rod and a saturated calomel electrode (SCE) were used for counter and reference electrodes, respectively. Using an aluminum slab (thickness 500 μm), EIS samples were prepared. Preparation of nanoparticle-deposited surface was described in Section 2. Except for the exposed area (1 cm^2^), the slab was masked using an epoxy resin and oven-dried at 95°C for 24 hours. An aqueous 3% wt NaCl solution was prepared for the electrolyte. A potentiostat (Voltalab PGZ40Z, Radiometer Copenhagen) was used for the EIS data aqcuisition. Before data acquisition, an individual test specimen was equilibrated in the electrolyte for five minutes. The scan frequency range of EIS was 10^2^ to 10^5^ Hz at a rate of 100 mV/s. The EIS data obtained from Nyquist plots was used to estimate capacitance in Eqs. (1) – (2).

### Pool boiling test

Pool boiling experiments were conducted by following the procedures reported previously^13^. Four T-type thermocouples (Omega, nominal uncertainty ± 0.5°C) were installed from the top along the axial heater direction at 6 mm intervals. A DC power supply (Agilent, DC5500) was used to control power to the cartridge heater. Voltage was increased by 1 V every five minute and allowed to reach steady state. Temperature was acquired utilizing a data acquisition system (IOtech). Vapor generated from the pool boiling chamber was condensed by three reflux condensers with a constant temperature controller (Polyscience).

## Author Contributions

B.J.Z., K.J.K. and T.H. designed and performed the experiment. T.H., J.D.N. and J.S. helped analyze the data. B.J.Z. and T.H. prepared all figures. The manuscript was prepared by B.J.Z., K.J.K., and T.H. with help from J.D.N. and J.S. All authors reviewed the manuscript.

## Supplementary Material

Supplementary Information Supplemental Information
