# Supplementary material for: Noncovalently assembled nanotubular porous layers for delaying of heating surface failure
Source: Sci Rep. 2014 Oct 29;4:6817. doi: 10.1038/srep06817 (PMC4212230; doi:10.1038/srep06817)
Supplement: Supplementary Information — Supplemental Information [file srep06817-s1.doc]

**Supplemental Information**

**Noncovalently assembled nanotubular porous layers for delaying of heating surface failure**

Bong June Zhang^1,2^, Taeseon Hwang^1^, Jae-Do Nam^3^, Jonghwan Suhr^3^, and Kwang Jin Kim^1,*^

^1^Mechanical Engineering Department, University of Nevada, Las Vegas, Nevada 89154, USA

^2^NBD Nanotechnologies, 8 saint Mary’s street, Boston, Massachusetts 02215, USA

^3^Department of Polymer Science & Engineering, Sungkyunkwan University 440-746, South Korea

*Corresponding author: [kwang.kim@unlv.edu](mailto:kwang.kim@unlv.edu)


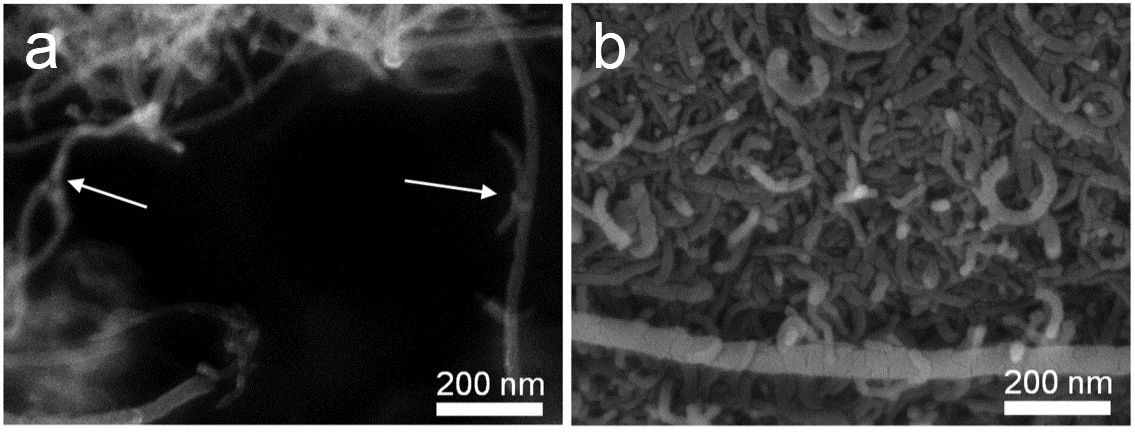


**Figure S1 |** **SEM images of FCNTs and PCNTs**: (a) White arrows indicate fused and/or entangled parts of FCNTs during functionalization of PCNTs. (b) PCNTs are aggregated by van der Waals interactions.


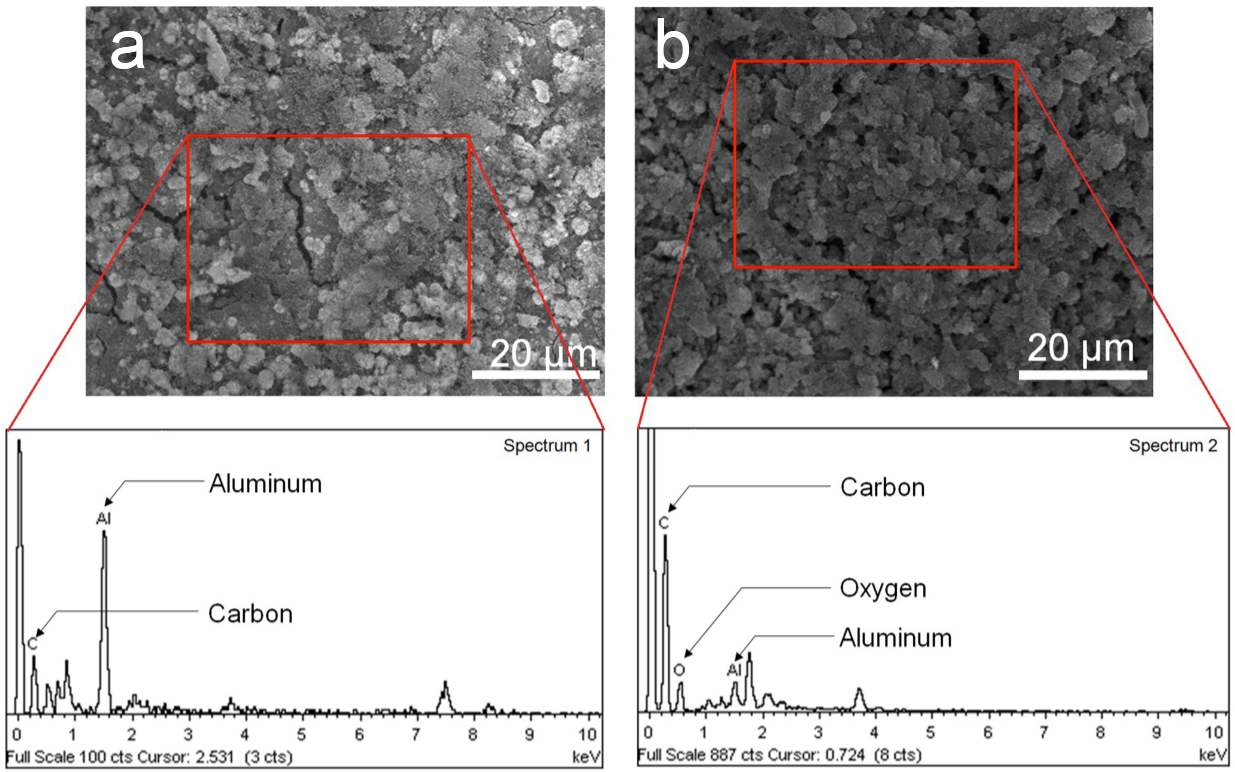


**Figure S2 |** **SEM images of nanofluids-deposited heating surfaces after pool boiling**: (a) PCNT at low magnification (x 500). Bare aluminum heating surface is shown on bottom left corner. Inset shows EDS spectrum of the selected area. Atomic peak ratio reveals Al:C = 87.9:12.1. Oxygen peak is relatively weak compared to carbon peak. and (b) FCNT at low magnification (x 500). Densely packed-porous agglomerates are shown. Inset shows EDS spectrum of the selected area. Atomic ratio shows C:O:Al = 75.4:23.1:1.5. Reduced aluminum peak indicates that the heating surface is thoroughly covered with FCNT aggregates.
